# Recent Advances on the Model, Measurement Technique, and Application of Single Cell Mechanics

**DOI:** 10.3390/ijms21176248

**Published:** 2020-08-28

**Authors:** Haibo Huang, Cihai Dai, Hao Shen, Mingwei Gu, Yangjun Wang, Jizhu Liu, Liguo Chen, Lining Sun

**Affiliations:** School of Mechanical and Electric Engineering, Jiangsu Provincial Key Laboratory of Advanced Robotics, Soochow University, Suzhou 215123, China; hbhuang@suda.edu.cn (H.H.); chdai@stu.suda.edu.cn (C.D.); 20194029003@stu.suda.edu.cn (H.S.); mwgu@stu.suda.edu.cn (M.G.); wangyangjun@suda.edu.cn (Y.W.); lnsun@hit.edu.cn (L.S.)

**Keywords:** single-cell mechanical model, micropipette aspiration, microfluidic, optical tweezers, atomic force microscope, disease diagnosis, regeneration and repair of biological tissue, micromanipulation

## Abstract

Since the cell was discovered by humans, it has been an important research subject for researchers. The mechanical response of cells to external stimuli and the biomechanical response inside cells are of great significance for maintaining the life activities of cells. These biomechanical behaviors have wide applications in the fields of disease research and micromanipulation. In order to study the mechanical behavior of single cells, various cell mechanics models have been proposed. In addition, the measurement technologies of single cells have been greatly developed. These models, combined with experimental techniques, can effectively explain the biomechanical behavior and reaction mechanism of cells. In this review, we first introduce the basic concept and biomechanical background of cells, then summarize the research progress of internal force models and experimental techniques in the field of cell mechanics and discuss the latest mechanical models and experimental methods. We summarize the application directions of cell mechanics and put forward the future perspectives of a cell mechanics model.

## 1. Introduction

The cell is the basic structure and a functional unit of an organism. It is the component unit of living bodies in the world. Cell size is extremely small, which needs to be seen under a microscope. In addition, the cell is also composed of many different molecules, so the cell is an important hub that connects macrolife and micromolecules [1]. In the past decades, biomechanics [2] has become one of the most active fields of scientific research. Biomechanics is an indispensable part of the foundation of modern medicine and life science. It is an interdisciplinary field and has many important applications in medicine, healthcare, life science, and biology. Cell mechanics is a frontier field of biomechanics and an important part of tissue engineering. The biomechanical properties of single cells are closely related to organs or macrotissues. Research shows that a change of single-cell size or morphology can affect the size and shape of organs [3]. Cell mechanics focuses on all kinds of cells in the human body, especially those related to the blood circulation system, the human body movement-support system, and the digestive system. Cell mechanics involves the study of the deformation of cell membrane and cytoskeleton, rigidity, viscoelasticity, adhesion force, and other mechanical properties of cells under mechanical loading, as well as the influence of mechanical factors on cell growth, development, maturation, proliferation, aging, and death. For disease research, the origin of many diseases can be considered from the mechanical properties of cells [4,5], such as the change of viscoelasticity of cells [6] and the change of Young’s modulus [7], which are related to cancer metastasis and malignant transformation.

In the wake of developments in science and technology, people are increasingly aware that cell mechanics plays a crucial role in cell function, and the experimental technology of cell mechanics has also been significantly changed. In addition, it also opens up a new way of research—mechanical models. A comprehensive understanding of the mechanical properties of cells will help us to further study living organisms. Therefore, the study of cell microstructure and other functional mechanisms is particularly important, and the establishment of a mechanical model of cell structure is also an important means of cell mechanics analysis [8].

Therefore, based on the cell mechanical model, this paper firstly introduces the classification and development of a single cell mechanical model. From the initial droplet model to the cell molecular dynamics model, these mechanical models have a good description of the mechanical properties and revealed the reaction mechanism of cells; however, these models also have their own shortcomings. Then, this paper introduces the common measurement technology of cell mechanical properties and the contribution and efforts of researchers using measurement technology. In addition, the application direction of cell mechanics is classified and explained. Finally, the future perspectives of the cell mechanical model are put forward.

## 2. Cell Mechanical Model

When establishing a cell mechanics model, not only the overall mechanical behavior of the cell but also the actual structure inside the cell must be considered, so that the model is more in line with the true state of the cell. The cell contains cell membrane, cytoplasm, nucleus, cytoskeleton, and other organelles, as shown in Table 1. When establishing cell models, we mainly consider the first four parts, which are responsible for the overall mechanical behavior of cells.

The cell membrane is the outermost layer of the cell, which is mainly a translucent membrane composed of phospholipids, with a thickness of 7–10 nm and bending rigidity of 10^−20^–10^−19^N·m [9]. The main components of the cell membrane are phospholipid bilayer, glycoprotein, glycolipid, and other substances. The phospholipid bimolecular layer is a two-dimensional fluid. When the temperature changes, the state of phospholipid molecules will change, realizing the mutual transformation of crystal state and liquid crystal state, and the fluidity of the cell membrane will also change. Cell membrane fluidity is a necessary condition to ensure normal function inside and outside cells. If the fluidity of the cell membrane is lower than a certain threshold, the activity of many enzymes and transmembrane transport will stop [10]. On the contrary, when the fluidity of the cell membrane is too high, the cell membrane will also dissolve. The main function of the cell membrane is to absorb, digest, and expel substances inside and outside the cell membrane through pinocytosis, phagocytosis, or exocytosis, so as to protect the balance of the internal environment.

Cytoplasm is the general name of all translucent, colloidal, and granular substances in the cell membrane except the nuclear area. The proportion of water is about 80% [11]. Its main constituents are ribosome, storage substances, various enzymes and intermediate metabolites, various nutrients, and monomers of macromolecules. The cytoplasm is the main place of life activities, which contains many organelles such as Golgi body, mitochondria, chloroplast (specific to plant cells), endoplasmic reticulum, lysosome, ribosome, and other substances. The cytoskeleton is located in the cytoplasm. The general concept of the cytoskeleton is a network system formed by the nuclear skeleton, the cytoplasmic skeleton, the membrane skeleton, and the extracellular matrix (ECM). In a narrow sense, the cytoskeleton is mainly composed of microfilaments, microtubules, and intermediate fibers. The cytoskeleton not only plays a vital role in preserving cell morphology and bearing external loads but also participates in many cellular life activities. Cells will have abnormalities in the cytoskeleton system under pathological conditions. In the process of malignant cell transformation, the depolymerization of microtubules will be greatly reduced [12], and the movement ability of cancer cells will also be enhanced. The nucleus is the most important substance in the cell, which is the regulatory center of cell genetics and metabolism. The nucleus mainly contains the nuclear membrane, chromatin, the nucleolus, and the nuclear matrix. There are thousands of nuclear pores on the nuclear membrane, which are distributed in the fusion of the inner and outer nuclear membrane, and these pores orderly regulate the transport and ion concentration of macromolecules inside and outside the nucleus. The significant substance in the nucleus is chromatin, which contains genetic information. Chromatin is the morphological expression of genetic material DNA and histone in interphase. When these substances are stimulated by external forces, they all bear certain mechanical behaviors.

When we establish the mechanical model, it is acknowledged that the mechanical behavior of cells is mainly determined by the cell membrane, cytoplasm, the nucleus, and the cytoskeleton [13]. According to statistics, there are nearly 100 mainstream cell mechanical models at present, but some models are only slightly improved or only explain part of the structure and function of the cell. There has not been much improvement in the overall cell mechanics model, so it is difficult to say that it is an independent cell structure model. In my opinion, for the composition of cells, it mainly includes the cell membrane, cytoplasm, the nucleus, and the cytoskeleton structure composed of microfilaments, microtubules, and intermediate filaments. Therefore, for the external mechanical reaction, it is mainly these parts that bear the main responsibility. Generally speaking, cell mechanical models can be divided into three categories by us: the continuous cortical membrane model, the discrete network cytoskeleton (CSK) model, and the cortical skeleton combined model. 

### 2.1. Continuous Cortical Membrane Model

The continuous cortical model regards cells as a kind of material with continuous properties, and the constitutive relationship and relevant parameters of the material model can be obtained through experiments. This method is simple, straightforward, and widely used, but at the same time, the cortical model also can help us understand the mechanical behavior inside cells. With more understanding and research on the cortical membrane model, many kinds of cell mechanics models have been derived, and the most common models are the four mentioned below.

#### 2.1.1. Droplet Model Surrounded by Continuous Elastic Cortex

Considering the whole mechanical behavior of the cell, an emphasis is placed on the membrane. The cell is reduced to a model surrounded by a continuous cortex, with a fluid-like sphere inside. This model shows that the cell has the duality of being solid and liquid, and the cortex is an outward layer tension that is used to maintain cell morphology. In addition, the liquid core inside the cell also has a certain viscosity. Evans and Yeung [14] treat the cell as a high-viscosity spherical droplet surrounded by a continuous elastic cortex. Evans performed microtubules based on a spherical droplet model and measured the mechanical properties of the cells.

Considering the mechanical properties of the whole cell, the spherical droplet model describes the real state of the cell, but it does not take into account the nucleus, cytoplasm, and other substances inside the cell, which has certain limitations. This model is suitable for the study of cell theory with large deformation and can predict the overall deformation and recovery of cells. However, when we use a pipette to suck in the cells too fast, the spherical droplet model cannot capture the immediate morphological changes of the cells, which is a limitation of the model. The prediction of cell mechanical behavior mainly depends on the mechanical parameters of the materials we choose, and different mechanical parameters can produce cell rheological behavior, which needs a lot of experimental calibration. However, the spherical droplet model also opens the door to the exploration of the cellular mechanical model.

#### 2.1.2. Solid Model

**Figure 1:**
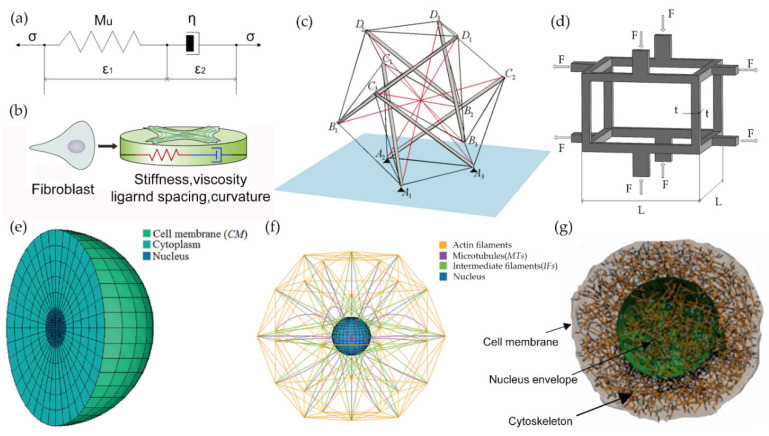
Cell mechanics model. (**a**) Maxwell model; (**b**) elastic model of fibroblasts (adapted by kind permission from [18]); (**c**) tensegrity mathematical model (adapted by kind permission of [30]); (**d**) foam model; (**e**) suspension cell mechanical model, continuum part (adapted by kind permission of [42]); (**f**) suspension cell mechanical model, cytoskeleton component (adapted by kind permission of [42]); (**g**) krill dynamics model (adapted by kind permission from [45]).

The solid model is a kind of model that is made up of a homogeneous solid substance or a variety of different solid substances. The main difference between this kind of model and the droplet model is that the center of emphasis of the model is transferred from the cell membrane to the internal environment of the cell, and the existence of cytoplasm and the nucleus is considered. The materials of this model are usually incompressible elastic solids or viscoelastic solids. The linear elastic solid model is the simplification and basis of the viscoelastic solid model. Hu [15] used a combination of the shear test and digital image correlation technology to measure the local viscosity of breast cells. Hu also used the generalized Maxwell model [16] to describe the local modulus and viscosity of cytoplasm and the nucleus to simulate the recovery of the time-varying creep effect of cells. Maxwell’s model is used to describe the viscoelastic characteristics of cells. As shown in Figure 1a, cytoplasm is connected in series with a damper through an elastic element, and then the large and small deformations of the cell are analyzed by the finite element method. Based on the biomechanical properties of articular cells and chondrocytes, Guilak [17] inquired about the biomechanical parameters of these cells and established a multiscale biphasic finite element model in the form of spherical inclusions. His research found that the force environment of cells changes all the time into a nonuniform form, and the mechanical properties of each part of the cells are quite different. Extracellular matrix and chondrocyte internal environments have different mechanical or biological characteristics, which will also affect the stress and strain in chondrocytes, indicating that extracellular matrix also has the function of biomechanics.

Paul [18] used the solid model to study the influencing factors of hepatocellular sclerosis and discussed the changes in cell morphology and mechanical behavior of fibroblasts in different external mechanical environments, as shown in Figure 1b. Mijailovich [19] and other researchers applied the model to finite element calculation. The linear elastic model is considered relatively simple, and it cannot fully describe the mechanical properties of the cell because the materials inside the cell have certain viscous characteristics and the properties of the viscoelastic material related to the loading rate and loading history. Schmid [20] established a homogenous viscoelastic solid model that regards the whole cell as a uniform viscoelastic solid. He used the parallel relationship between the Maxwell model and the spring to obtain the Kelvin model [21] (standard linear solid model) for simulation. Studies have shown that this model is suitable for both osteoblasts and chondrocytes [22].

#### 2.1.3. Damping Model of Power Series Structure

The solid model is mainly obtained from the instantaneous loading conditions. However, cells will be affected by dynamic forces in their physiological environment. The dynamic storage modulus and loss modulus in the model can describe mechanical behavior under the dynamic forces. Unlike the previous model, this model is a frequency-domain model. This model can effectively describe the mechanical behavior of adherent cells. Alcaraz and Fabry [23,24,25,26] have established the constitutive relationship of the model, which can explain the dynamic mechanical behavior of various adherent cells. Hence, the model is suitable for the smooth muscle cells of the human body, the epithelial cells of the lung, and neutrophils.

#### 2.1.4. Two-Phase Model

All the models above regard cells as single-phase materials, while cytoplasm is composed of solid polymer and fluid, so it is more practical to treat cytoplasm as a two-phase material. Compared with the previous models, the theoretical complexity and irregular geometry of cells make the analytical solution difficult to obtain. Shin [27] simulated the experiment with the finite element method and applied the mechanical parameters of the two-phase model to the calculation. 

It is generally believed that the continuous cortical model of cell structure can correctly reflect the behavior of cells under stress to a certain extent, for example, the viscosity property, elastic property, and stiffness property of cells. In particular, the mechanical behavior predicted by these models can also be consistent with the actual measurement value in many cases. However, such models take too much account of the role of the continuous cortex of cells and ignore the role of microfilaments, microtubules, and intermediate filaments under the cell membrane, as well as the role of the micronetwork woven by these microfilaments, microtubules, and intermediate filaments. Therefore, this kind of model cannot be consistent with the cell’s actual network structure, nor can it completely reflect the whole mechanical behavior of cells under stress.

### 2.2. Discrete Network Skeleton Model

The discrete network skeleton model of cell structure focuses on the role of microfilaments, microtubules, and intermediate filaments under the cell membrane, as well as the network woven by them. This model believes that the behavior of cells under stress is mainly determined by the microfilaments, microtubules, and intermediate filaments under the cell membrane and their micronetworks. Microtubules play a major role in resisting external forces, and microfilaments are mainly responsible for the tension of cells. Intermediate filaments play the role of connection. These microstructures increase the strength of the network. With the continuous exploration and research of investigators, the skeleton model of the discrete network has changed a lot.

#### 2.2.1. Tension Integration Model

The tension integration model is one of the most typical models, which was proposed by Harvard scholar Ingber [28,29]. This model is mainly based on the discrete network structure of microfilaments, microtubules, and intermediate filaments under the cell membrane. It is believed that the mechanical properties of cells are mainly determined by the properties of these networks, just as the steel structure skeleton of a building determines the mechanical properties of the building. They began by using some wooden sticks and rubber bands to make various models of tension integration cells. On this basis, Sultan [30] proposed a spherical tensegrity structure with intermediate fibers, and the tensegrity model used to generate the calculation is shown in Figure 1c. Tendons represent microfilaments (black thread) and intermediate filaments (red thread). Thick gray pillars represent microtubules. The anchor point (blue) of the base material is represented by black triangles (A1, A2, and A3). The key to the tension model is the prestressed structure of the skeleton, which is the basis for the cell to maintain an equilibrium state. Wang [31] established a 3D tension finite element model of adherent cells to simulate cell origami (using the pulling force of living cells as the biological driving force to fold various three-dimensional cell-filled microstructure technology) and studied the influence of the complexity of tension structure on the folding angle of microporous plates; the complex calculation model can more effectively reflect the research of mechanical conduction mechanisms inside cells.

The tensegrity model has a good explanation for the biochemical response of external mechanical stimulation [32]. This model has successfully proved some basic characteristics of cells under stress, such as the linear relationship between stress strength and cell stiffness and cell migration behavior [33]. However, this model also has some obvious defects. For example, if you remove one of the rubber bands or wooden sticks, the whole model will collapse, which is not consistent with the real situation of the cell. In view of the defect of the collapse of the whole model caused by cutting off a rubber band or wood bar, it has been proposed that the rubber bands and wood bars of the model should be randomly distributed. In addition, these elements should be given a lot of redundancy in any possible stress direction so as to avoid the whole collapse of cells caused by taking away a rubber band or wood bar. However, there are some problems in this theory; for example, the tissue distribution in cells is not really random because we all know that the tissue of cells achieves a specific function, so the tissue distribution of cells cannot be completely random. In addition, it is impossible to have so many redundant tissues in cells. There is a saying in the world of biology that “useless is useless”, [34] and cells must also survive the situation of evolution. Even if there is tissue redundancy in cells at the beginning, with the evolution and development of cells, redundant tissues in cells will disappear, which is in line with the law of biology.

#### 2.2.2. Porous Solid Model

This model mainly observes that the filamentous network structure of the cell is specifically shaped like the geometry of foam, so this model can also be called the foam model. The foam model is a network model connected by elastic rods, as shown in Figure 1d.

The two key parameters of the model are relative foam density φ (the ratio of the volume of a single member to the volume of foam) and Young’s modulus of a single rod $E_{f}$. The relative foam density can also be obtained from the ratio of foam mass density to the mass density of a single rod. The elastic modulus of the structure depends on the tension or bending of the member and is proportional to φ or $\varphi^{2}$. The main ways of restoring stress in response to external loads are tension, bending, and torsion. Satcher [35] used this model as the actin cytoskeleton (CSK) model of endothelial cells and assumed that the bending and twisting of microfilaments are the main mode of stress generation in the actin grid. The strain hardening characteristics predicted by the continuous bubble model are consistent with those of the adherent cells under local pressure, which is reasonable. However, the foam model simplifies CSK actin fiber into a rod, ignoring the different mechanical properties of filaments, microtubules, and intermediate fibers.

#### 2.2.3. Cable Net Model

Coughlin and Stamenovic [36] proposed a cable net model in which the cytoskeleton is assumed to be an ideal articulated elastic cable net. In this model, the microfilament is assumed to be an elastic wire rope and can only act as a pull force, connecting the microfilaments by means of an articulated joint. The main feature of the cable network model is to assume that the discrete units constituting the cytoskeleton are the same force units, which constitute a regular network structure. However, in reality, it is impossible to have the same structure of mechanical parameters (elastic modulus) and size (radius). The different structures that make up the cytoskeleton, such as microfilaments, microtubules, and intermediate fibers, will have differences and need to be treated differently.

#### 2.2.4. Biochemical Mechanical Model

Deshpande [37] first proposed a biochemical mechanical model (BCM) of cell contraction to explain the dynamic recombination behavior of the cytoskeleton. The BCM model links the biochemical behavior in cells with the tension and contraction force of stress fibers so that we can have a deeper comprehension of the biochemical process of cells. The BCM model defines a single stress fiber that contains three key biochemical processes: (1) an activation signal to promote protein polymerization and myosin phosphorylation; (2) myosin and actin are assembled into stress fibers through tension; (3) tension is generated by the cross-bridge cycle between actin and myosin; this model contains the biochemical behavior in cells, which can explain many mechanical behaviors in cells. Truong [38] used the biochemical mechanical model to study the generation process of actin in osteoblasts. Osteoblasts were placed in different sizes of titanium microarray to predict the distribution of actin in cells. The biggest advantage of this model is to explain the dynamic reorganization of the cytoskeleton in cells. Through Hill’s fiber contraction model, the interaction between myosin and actin can be better understood. The biochemical mechanical model can predict the effect of compliance on cell traction and the dependence of cell size and force. Furthermore, it also can analyze the influence of boundary conditions, cell shape, and external force on the location of stress fibers and the distribution of adhesion spots. Although the biochemical mechanical model can provide a lot of mechanical information, it still needs to be improved. In terms of cell composition, there are a large number of different substances in the cell, such as mitochondria and lysosomes, and they are all responsible for the overall mechanical behavior. Therefore, more complex constitutive equations that can simulate all substances in the cell need to be proposed so as to better study the overall mechanical behavior of cells.

### 2.3. Cortical Skeleton Combined Model

To a certain extent, the two models above have a partial explanation and description of the mechanical behavior and biological response of cells, but they both have certain limitations. For example, the continuous cortical membrane model simply assumes that cells are composed of continuous cortical layers, without considering the contribution of microfilaments, microtubules, and intermediate filaments to the mechanical behavior of cells, while the discrete network skeleton model, similarly, does not consider materials such as the membrane, cytoplasm, and the nucleus. The cortical skeleton combined model combines the features of the two to form a new cell mechanics model.

#### 2.3.1. Cortical Skeleton Combined Model Based on Finite Element Method

Cells and their extracellular environment interact with each other, and we have no complete understanding of the reaction mechanism of external mechanical stimulation and the mechanism and principles of the biochemical reaction. McGarry [39] proposed a finite element model of adherent cells that combines the cell components in the structure, including the prestressed cytoskeleton, cytoplasm, nucleus, and cell membrane. This model is a combination of the solid model and the tensegrity model. ANSYS (simulation software) can be used to effectively analyze the contribution of each component of cells to the overall mechanics of cells. It is found that the cytoskeleton plays a decisive role in the stiffness of cells, and the cytoplasmic, nuclear, and other cell groups also have a certain impact on the stiffness of cells. Feng [40] created a single cell finite element model that combines the tension model with the continuous cortex model. The model includes the cell membrane, cytoplasm, microtubules, intermediate filaments, and the nucleus. In order to verify the model, the finite element method was used to simulate the cell indentation experiment of AFM, and the comparative analysis results were basically consistent with the experimental data. By changing the elastic modulus of the cell membrane and the density of skeleton microtubules and intermediate filaments to simulate cell aging (with the increase of age, the actin density of animal cells decreases, which reduces the element density of the cytoskeleton), combined with an indentation experiment, the mechanical response of young cells and aging cells under the stimulation of external forces were analyzed. The results showed that there is little difference in mechanical properties between young cells and old cells. Through the skeleton cortex combination model, cell mechanics could be predicted with the growth of age changes in characteristics. To analyze the effect of the curvature of the substrate on the mechanical properties of adherent cells, based on the tension theory, Vassaux [41] established a mechanical model containing the cell membrane, the skeleton microfilament network, microtubules, the nuclear model, and the nuclear skeleton. It was found that the more concave the substrate, the more stable the nuclear structure. 

Deepak [42] proposed two finite element models to simulate the mechanical deformation of cells under different external forces. One is the suspension cell mechanics model, which includes the continuum (Figure 1e) and the discrete cytoskeleton (Figure 1f). The model simulates the state of compression and stretching in microtubules and obtains the curve of force and elongation. Taking smooth muscle cells as experimental objects, the data of force and elongation of cells during stretching are obtained. The two curves are basically the same. It has been found that when cells are stretched, the reaction force of cells increases nonlinearly with the increase in elongation. The other is the adhesion cell model, which is similar to the mechanical model of suspension cells. Taking adherent embryonic stem cells as the experimental object, the cell indentation curve simulated by this model is basically consistent with that of embryonic stem cells. The simulation results show that the actin filaments and microtubules of the cells play a very important role when the cells are stretched. In the indentation experiment, the actin cortex, actin bundles, and microtubules are responsible for the stiffness of the cells. Sara [43,44] used ABAQUS to simulate the indentation experiments of single adherent cells and single beads. He established the three-dimensional finite element single-cell model, which contains different components such as cytoplasm, nucleus and cytoskeleton, and actin and microtubule elements. This model collects information about how cells respond to forces and deformations under pressure and how forces are transmitted to a single frame through CSK components and cytoplasm. Subsequently, finite element cell models have been used to investigate the response to different mechanical stimuli, as well as which components of CSK are primarily responsible for changes in the external load. The results show that different CSK elements have different mechanical responses to specific external disturbances in the deformation process. It was founded that the actin cortex and microtubules were responsible for anticompression, and the actin bundles and microtubules were key to antishear loading.

To some extent, the finite element method of the combination model has a good explanation for the specific mechanical behavior of cells. However, the cells will always have a biochemical reaction in the real environment, and the cytoskeleton will have a continuous polymerization and depolymerization reaction. The microfilaments, microtubules, and intermediate fibers in the finite element model are all composed of materials with specific constitutive equations; this is not very consistent with the real situation of cells and has certain limitations.

#### 2.3.2. Cortical Skeleton Combined Model Based on Molecular Dynamics

Molecular dynamics is a molecular simulation method that mainly relies on Newtonian mechanics to imitate the movement of the molecular system. The cortical skeleton combined model, united with the molecular dynamics method, can explain the macromechanical behavior of cells and give a better understanding of the mechanism of cell mechanical response at the molecular level. The dissipative particle dynamics (DPDs) [46] in molecular dynamics is a new simulation method in the field of colloid and interface science. It can simulate the dynamics and rheology of polymers in solution, the kinetics of microphase separation in block copolymer melt, and the effect of hydrodynamics in spinodal decomposition. Liu [47] proposed dissipative particle dynamics to establish a cell dynamics model, including a cell membrane network, the cytoskeleton, cross-linking proteins, and motor proteins. Kirill [45] proposed a new eukaryotic cell model based on mesoscopic particles that clearly describes the cell membrane, nucleus, and cytoskeleton, as shown in Figure 1g. The dissipative particle dynamics (DPDs) method provides us with a new method to simulate the cell and its interaction in the flow. The contribution of subcellular components to whole-cell mechanics in the experiments of microtubule aspiration and microfluidics was discussed. MCF-10A cells were used as experimental objects. Firstly, the model parameters were calibrated by microtubule aspiration simulation, and then the model was verified using the data obtained by the medium-sized cells flowing through the microfluidic device. The parameterization of the cell model is flexible and can be used to simulate various types of cells.

The molecular dynamics model has opened the door for us to explore the real microstructure of cells and also effectively expanded the depth of cell mechanics model research. However, this kind of model needs us to have good research on biomolecules. The simulation of biomacromolecules is very complex, and the amount of data is huge. The simulation speed is slow, and the simulation of the real environment of cells is also different. The following is a summary of all models, as shown in Table 2.

## 3. Research Progress in Experimental Measurement Techniques of Cell Mechanics

The cell mechanical model is closely related to the development of mechanics measurement technology, which has also been greatly developed. Based on the analysis of biomechanical experiment technology, it can provide a good research platform for cell mechanics models and greatly promotes the development of cell mechanics models. For measurement technologies, the main mechanical parameters are shown in Appendix A. These mechanical parameters are not completely independent; all have their internal relationships. Viscoelasticity, for example, is a combination of elasticity and viscosity. The viscoelastic materials have both the viscosity of a fluid and the elasticity of a solid. In addition, taking Young’s modulus as an example, it is a mechanical parameter specially used to describe the resistance to deformation, which is used to describe the elastic capability of materials. Common techniques include micropipette aspiration (MPA), microfluidics, optical tweezers, and atomic force microscopy (AFM). The characteristics of the various measurement techniques are shown in Appendix A Table A2 [48,49,50,51].

### 3.1. Micropipette Aspiration Technique

**Figure 2:**
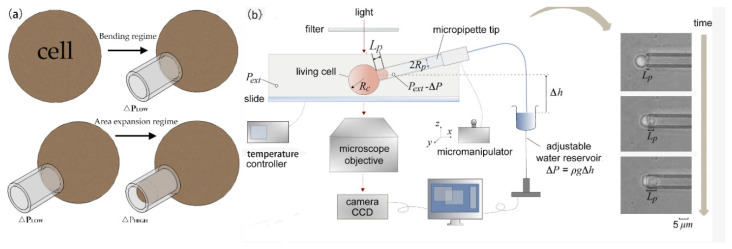
(**a**) Micropipette aspiration schematic diagram. (**b**) Experimental scheme and images of micropipette aspiration. (Adapted by kind permission from [65]).

Micropipette aspiration is a common experimental technique used to measure the mechanical properties of single cells. A micropipette aspiration schematic diagram is shown in Figure 2a. This technique was first proposed by Mitchison and Swan to study the mechanical properties of the cell surface. Experiment devices are usually composed of a micropipette, a pressure pump, a CCD camera, a micromanipulator, and a microscope. Negative pressure is released by the pressure pump, and some or all of the experimental cells are inhaled into the microtubule, as shown in Figure 2b. Rand [52] first used this technology to measure the elastic modulus and shear modulus of the red blood cell membrane. Cheng [53] simulated the process of separating red blood cells (RBCs) on the flat matrix and introduced the diffusion model describing the migration of receptors in the cell membrane; the simulation results fit the experimental data well. Later, Shao [54] optimized micropipette aspiration technology by computer optimization data analysis. The applied pressure has a linear relationship with the length of the aspired cells:

(1) $$∆p=\frac{2\pi }{3}E\frac{L}{a}\varphi$$

In the above formula, E represents Young’s modulus of the cell. L and a indicate the inner radius of the micropipette and the inhaled length of the cell, respectively. φ describes the wall function, and ∆p is the suction pressure.

Micropipette aspiration technology is used in the study of adhesion characteristics of soft materials [55,56,57], and the study of cells and biological tissues [58] is also very popular. Wendy [59] used micropipette aspiration technology to study the adhesion characteristics and Poisson’s ratio of chondrocytes and verified the viscoelastic mechanical model. Wendy simulated the experimental process of osteocyte aspiration by using finite element technology. Li [60] analyzed the influence of cell size and compressibility on the process of micropipette aspiration and characterized the creep behavior of chondrocytes. Kristina [61] evaluated the effect of lifeact GFP (green fluorescent protein) and actin GFP on the mechanical properties of cells by using micropipette aspiration technology. Taking bone marrow stem cells as the experimental object, combined with the linear elastic solid model, it was found that lifeact GFP had little effect on the equilibrium modulus and instantaneous modulus of cells, hardly changed the mechanical properties of cells, which made it more convenient for us to observe and study the mechanical properties of cells. Hogan [62] used micropipette aspiration technology to characterize the adhesion characteristics of cells and studied the effects of different geometric parameters on mechanical behavior, such as adhesion area, loading rate, diameter of pipette, and also the critical separation force of cells. With the further study of the mechanical features of the cell surface, it is believed that the skeleton and nucleus in cells also have certain mechanical properties. Guilak [63] measured the viscoelastic properties of the nucleus by micropipette aspiration and verified the viscoelastic mechanics model. In addition, in order to study the stress transfer mechanism of the nucleus during cell deformation, Ashkan [64] developed a mathematical model that consists of two solid models (the Maxwell model and the linear elastic mechanics model), assuming that the nuclear membrane and nucleoplasm constitute the nucleus. He used micropipette aspiration technology to study the force and deformation of the nucleus during aspiration.

In the research process of cell mechanics, micropipette aspiration technology has been favored by many researchers. There are many new methods based on micropipette technology. In different kinds of cells, the measurement of mechanical properties is widely used [65]. Gerecsei [66] used a computer to control micropipettes so as to achieve high-throughput measurement of the surface adhesion characteristics of particles. Hirano [67] established a feedback control system by combining scanning electron microscopy and micropipette technology. The signal detected by SEM (scanning electronic microscopy) can regulate the transmission of MP solution so as to realize the quantitative transmission of MP solution in cells. Ankita [68] combined ion current technology with MPA technology, obtained the cell deformation through the ion resistance at the tip, and measured the mechanical capabilities of red blood cells by the ion current derivation. In a 0.7-μm pipette, potential cell rupture was identified by electrical signals. Daza [69] measured the elastic modulus of lymphocytes with AFM and MPA, respectively, and found that the experimental data obtained by the two had a three-fold difference. The reason for the difference was analyzed, and it was considered that the cell adhesion characteristics and the nonuniformity of the internal skeleton affected the overall mechanical properties of the cells. Alireza [70] used micropipette aspiration technology, combined with confocal technology, to obtain Young’s modulus and viscosity characteristics of human umbilical vein endothelial cells in order to study the changes in the mechanical performances of cells under single electric radiation. It was found that the hardness of cells increased significantly, and the creep flexibility curve decreased significantly under the effect of electric radiation. In general, micropipette technology is widely used because of its high precision, good repeatability, and relatively low cost.

### 3.2. Microfluidic Technology

Microfluidic technology can be applied in cell biology, disease research, and drug development [71,72,73]. Microfluidic technology can provide us with a lot of mechanical information (shear stress, constraint, base stiffness) [74]. Microfluidic technology is based on the analysis of the deformation of cells in the process of microchannel crossing so that the mechanical information can be obtained. Microfluidic technology has a very high precision, which can reach the nanometer level of distance. In addition, it has a high degree of automation and can repeat high-throughput experiments [75,76]. The interaction between cells and the surrounding environment will lead to a series of mechanical regulation of cells. For the mechanical characteristics of the interaction between cells and the extracellular matrix, microfluidic technology can effectively measure the displacement information of cell activities and the traction between cells and the extracellular matrix [77].

**Figure 3:**
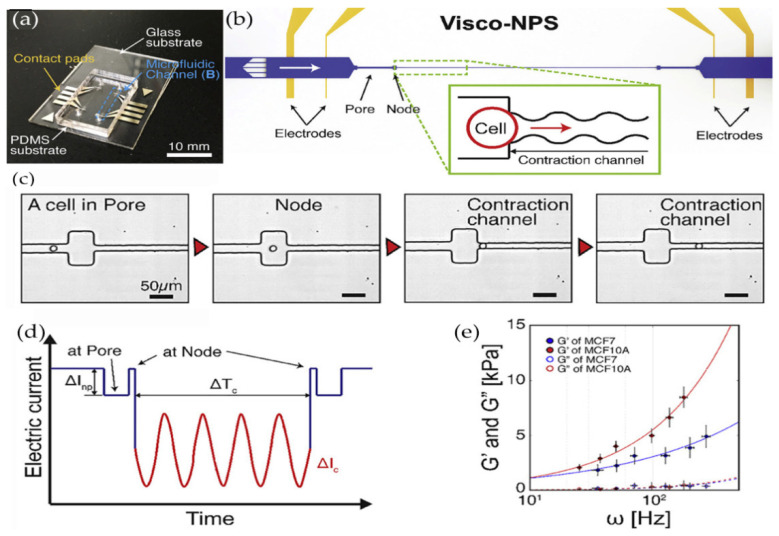
(**a**) The schematic diagram of the microfluidic channel. It has three main components: a pore, a node, and a sinusoidal contraction channel (which is in the green dotted box). (**b**) By applying DC voltage to the cnnel in the external electrode, the channel current can be measured from the internal electrode pair. (**c**) Cell snapshots in different regions of the microchannel; (**d**) the expected current pulses generated by cells through the microchannel; (**e**) the storage modulus G′ and loss modulus G″ of MCF-7 and MCF-10A cells. (Adapted by kind permission from [88])

Wang [78] designed a computational model, combined with the herringbone structure in the microfluidic device, to track the movement of cells so as to achieve the capture and detection of cancer cells. Fan [79] established an impedance measurement analysis system that can be used to detect the osteogenic differentiation of bone marrow stem cells at any time and designed a single cell measurement microfluidic chip that can efficiently capture and precisely locate single cells. Dweep [80] used two sets of microfluidic devices (the hydraulic pipette device and the extrusion device) to change the force of cancer cells and measure the deformation ability of cells. In addition, two sets of devices connect the deformation force with surface rheology to verify whether there is a certain protein in cells related to the deformation ability of cells. Ye [81] studied the relationship between the time of cells passing through the microchannel and the mechanical information of single cells passing through the microchannel, established the relational expression between the passing time and cell shear modulus and bending modulus, and studied the relationships between the flow rate and cell deformation in the microfluidic chip. Alapan [82] used the shear stress of water flow in the microfluidic device and cell size algorithm to analyze the dynamic deformation ability of adherent red blood cells. Guillou [83] designed a new cross slot microfluidic system that uses hydrodynamic force under a low Reynolds number and low restriction, which stretches cells near the stagnation point and measures the viscoelasticity of fiber cells and tumor protocells. The results are in good agreement with the data of the aforementioned micropipette aspiration technology. Solis [84] designed a novel microfluidic nano biosensor in the microfluidic channel to observe the adhesion characteristics of fiber cells in cell culture. Ji [85] designed a microfluidic cell meter based on double photodiodes and analyzed the influence of light intensity on the change of cell mechanical behavior. In addition, different cells were distinguished by the size of transmitted light intensity. Spuul [86] designed a microfluidic device to study the actin cytoskeleton in a narrow space, which can be used to analyze living cells in a closed space with a fluorescence microscope. Soffe [87] designed a trapezoid microchannel to generate a transverse shear stress gradient so as to analyze the mechanical response of cells in the channel and the effect on the intracellular calcium signaling pathway. Kim [88] used a sensor with a bonding hole in the microstereo platform, as shown in Figure 3. He measured the storage modulus and loss modulus of mammary epithelial cells, respectively, so as to quantify the viscoelastic difference between benign cells and malignant cells. The application and development prospects of microfluidic devices in cell mechanics are very broad, and the microfluidic devices are highly modular so they can perform multiple functions in a compact device. In the future measurement of cell mechanical properties, the advantages of microfluidic devices will become more and more obvious.

### 3.3. Optical Tweezer Technology

**Figure 4:**
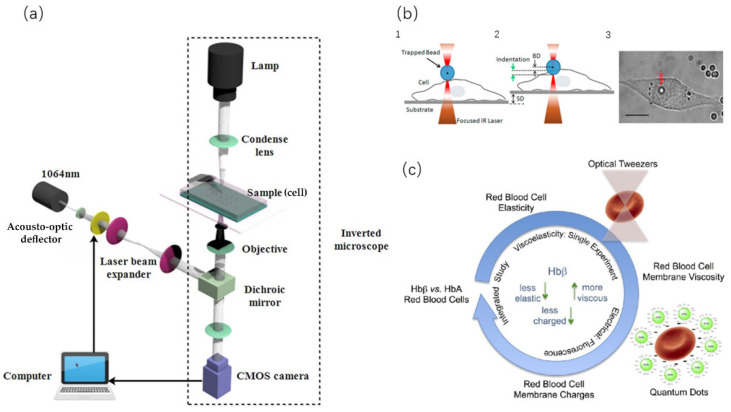
(**a**) Experimental devices of optical tweezers; (**b**) schematic diagram and experimental image of indentation experiment; (**c**) measurement of viscoelasticity and membrane charge of erythrocytes. (Adapted by kind permission from [112]).

Optical tweezer is a vivid description of a single-beam gradient force optical trap. As they are similar to mechanical tweezers, they have the function of manipulating objects. The experimental devices of optical tweezers and a schematic diagram are shown in Figure 4a. Compared with the traditional micropipette aspiration and AFM technologies, the optical tweezers have little influence on small objects because they do not form mechanical contact with the target to be measured. Optical tweezers are widely used in many fields, such as nanofabrication [89], DNA biology research, and cell micromanipulation [90]. In addition, because the cell membrane is transparent, the light beam of optical tweezers can pass through the membrane and control the cell interior, which is an advantage that other experimental techniques cannot achieve.

Optical tweezers control, manipulate, and measure the mechanical information of the cell surface by focusing a light beam. The measurement force can reach the level of pN [91,92]. Ashkin [93,94] first used infrared beams to manipulate cells, viruses, and other organisms and successfully demonstrated capturing, moving cells, and manipulating the internal structure of cells such as organelles. Xiang [95] integrated automatic capture and manipulation into a single unit and proposed a closed-loop control method to realize the dynamic capture and manipulation of cells. Yan [96] combined optical tweezers with a microfluidic device to realize femtosecond optical tweezer movement and cell-sorting in the microfluidic device. When optical tweezers capture cells, there is a certain requirement for the location of cells; that is, the cells must be in the optical trap. Li [97] designed a controller for cell capture and manipulation that can limit cells to the local area around the optical trap and ensure the distance between yeast cells and the surrounding obstacles. In addition, Li [98] also proposed a new high-speed manipulation method using robot-assisted optical tweezers that can manipulate cancer cells of human leukemia to specific areas so as to achieve accurate measurement. Cao [99] used holographic optical tweezers to rotate a single mammalian cell. This method can realize a 360° rotation in a spherical or nearly spherical cell 3D space. Zhang [100] proposed an optical tweezer that is based on hollow annular-core fiber (HACF) that can realize the operation of living cells and aseptic transportation. Isaac [101] used a circular beam to manipulate swimming cells in order to achieve directional operation. Seeger [102] combined optical tweezers and a light knife to cut and realize accurate operation on cells.

All of the abovementioned techniques use optical tweezers to precisely and directionally manipulate cells at high speed. Now, we can focus on the measurement of the mechanical parameters of cells [103]. For the first time, Henon [104] measured the shear modulus of the membrane of red blood cells by using optical tweezers. After binding two small silicon beads on the cell membrane, he captured and stretched cells by applying calibrated tweezers so as to obtain the shear modulus of red blood cells (2.5 ± 0.4 µN/m). Muhammad et al. [105,106] measured the local elasticity of cells by squeezing and stretching them with the indentation method, as shown in Figure 4b. In addition, Muhammad [107] also used optical tweezers to explore the influence of the basal region on the hardness of cancer cells. Lee [108] used self-made optical tweezers to measure the aggregation and decomposition of interacting red blood cells. Wu [109] also proposed a new method to calibrate the trapping force and drag force on living cells. Liu [110] studied the mechanical properties of red blood cells after oxidation and measured the shear modulus of red blood cells after H_2_O_2_ treatment. Alex [111] employed optical tweezers to study the adhesion force between malaria protozoa and red blood cells. Once the adhesion was reduced, the malaria protozoa’s invasion of red blood cells was also inhibited. Carinna [112] combined optical tweezers and cation quantum dot (QD) technology to measure the membrane charge and viscoelasticity of red blood cells, as shown in Figure 4c. Yu [113] combined optical tweezers and stretchers to explore the viscoelastic properties of cells and built a spring damping model (Maxwell model) and a power-law model (damping model of power series structure) to obtain the information on creep properties. Tan [114,115] used optical tweezers to stretch red blood cells and obtained the relationship between force and deformation. On the basis of previous work, combined with the two-phase model under the condition of low permeability, the simulation results were in line with the experimental data, and results showed that the cell stiffness increased with the osmotic pressure. Bareil [116] proposed a numerical method based on linear elastic theory to calculate the shape deformation of spherical cells from the distribution of photonics stress on the cell surface. Optical tweezers can realize noncontact control and detect the mechanical properties of cells, but for cells that are easy to deform at any time, the calculation of force and torque of optical tweezers is also a big challenge [117].

### 3.4. Atomic Force Microscope

**Figure 5:**
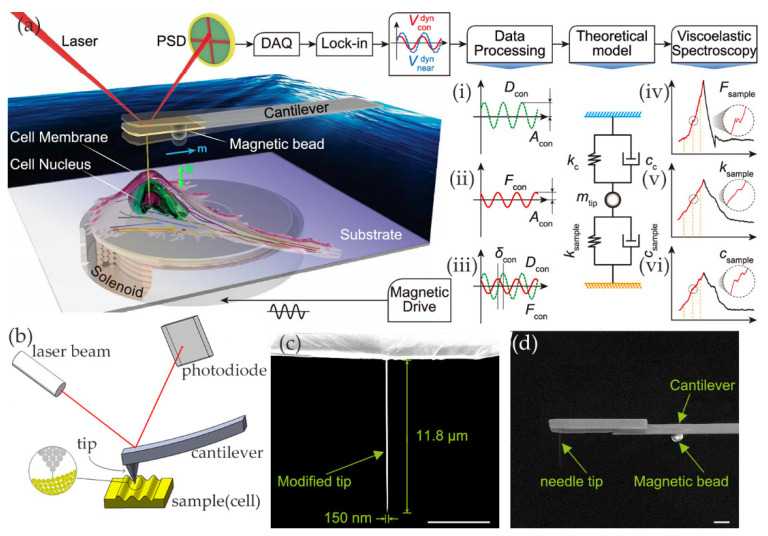
(**a**) Experimental model diagram, signal processing flow, and spring-mass model. (i) to (vi) represent dynamic mechanical phenotypes of cells. (**b**) AFM schematic diagram. (**c**) AFM tip made of pyramid tip etched by focused ion beam (FIB). (**d**) Improved AFM probe which contains three parts: micromagnetic bead, needle tip, and cantilever. (Adapted by kind permission from [144]).

An atomic force microscope is a technology with high measuring resolution and a broad range. This technology uses a small tip on a flexible cantilever to contact the object to be measured, and the measuring precision can be from µN to pN. AFM is a kind of analytical instrument that can analyze the surface structure of solid materials. Its core function is to scan the surface of an object and obtain a three-dimensional image of the object’s surface [118]. AFM includes the tip of a needle, a microcantilever, a motion detection device, a feedback circuit, a scanning device, and an image collector, as shown in Figure 5b. When the tip gets close to the sample, a repulsion or attraction force between them is generated and measured so as to obtain the true appearance of the surface with nanometer resolution. The stress–strain curve of cells can be obtained by AFM, and even the cell dynamics and cell movement can be detected in real-time.

AFM can be used to measure the mechanical parameters of various biological samples, such as elasticity and adhesion [119]. So far, AFM has been applied to many kinds of cells, including epithelial cells [120,121,122], fibrocytes [123,124], cancer cells [125,126,127,128,129,130,131,132], hematopoietic stem cells [133,134,135,136], yeast cells [137], and somatic cells [138]. Bastian [139] observed the elastic behavior of epithelial cells with AFM. Guo [120] studied the elastic properties of different regions of human aortic endothelial cells (HACE) and the overall mechanical information of the cells by AFM. Codan [123] used mouse fibroblasts and human epithelial cancer cells as experimental objects and studied the elastic difference between living cells and fixed cells by AFM. Nikolay [133] measured the stiffness change of stem cells during the apoptosis process with an atomic force microscope. Ida [134] used AFM to observe the effect of carbon-coated iron nanoparticles on the elastic modulus of human mesenchymal stem cells. Zemla [140] used atomic force microscopy to evaluate cell elasticity and adhesion to identify cancer cells and tissues. Hayashi et al. [125,126,141] used the AFM indentation method to analyze the hardness of cancer cells. Li [142] used a microsphere probe to measure and distinguish benign and cancerous human breast epithelial cells. The elastic behavior of cells is affected by the external environment. Previous studies have reported the difference between cancer cells and normal cells. The measurement of the mechanical behavior of cancer cells is mainly focused on one parameter—Young’s modulus [143]. However, it is still controversial whether Young’s modulus can describe the severity of cells.

The abovementioned AFM mostly analyzes the elastic behavior of cells, and the measurement of cell adhesion force to the external biological environment is also very common. Ryu [145] measured cell adhesion with an arrow nanoneedle and AFM. Kim [130] used atomic force microscopy to quantitatively measure the intercellular adhesion between macrophages and cancer cells. Li [146,147] used AFM to measure the elasticity of living cells. Tanmay [141] studied the effects of cell cycle and probe geometry on the mechanical properties of pancreatic cancer and quantitatively analyzed cell–matrix interaction by AFM. Codan [124] used the indentation method of AFM to explore the adhesion behavior of fibroblasts. In general, the indenter of AFM is the tip. Martin [148] changed the tip of AFM into a wedge-shaped structure (parallel plates that compress cells), which reduced the lateral displacement of cells in the measurement process. In addition, Nadja [149] combined atomic force microscopy with acoustic probe cells. Laura [150] used a planar AFM macro probe to study the mechanical properties of cells larger than 100 μm in diameter. Rother [151] used the microrheology experiment, combined with an atomic force microscope, to discuss the mechanical properties of breast cells under different metastatic potentials and quantified malignant tumors’ mechanical parameters. He proposed a model-free method based on monitoring loss tangents, which represent the ratio of loss modulus to storage modulus of the detected unit. The study found that cancer cells showed greater tangent loss than benign phenotypes, which means that the fluidity of cancer cells is higher than normal cells. Choi [152] studied the biomechanical changes of single cells induced by nonthermal atmospheric pressure microdielectric barrier discharge plasma. He used AFM to measure the force distance curve. In addition, combined with Raman spectrum observation, it has been found that the chemical changes caused by cancer cells after plasma treatment are much higher than that of normal cells. When cells are stimulated by discharge plasma, the amount and organization of actin in cancer cells will change, and the mechanical properties will be more obviously reduced. Jens [153] used atomic force microscopy and single-cell force spectroscopy (SCFS) to measure the adhesion strength of various animal cells to matrix. Xie [144] combined the viscoelastic spectrum and atomic force microscope to study the internal structure of cells, as shown in Figure 5a. They designed a magnetically driven nanoneedle and quantified the viscoelastic spectrum of the internal structure of cells through the amplitude and phase of the needle tip. The diameter of the needle tip is 150 nm, as shown in Figure 5c,d. They extracted the multidimensional mechanical phenotypes for three different types of cells (Hela, SiHa, and NIH3T3). Each of the three types of cells has a specific mechanical phenotype; through the analysis of its mechanical phenotype, they could effectively distinguish cancer cell lines. Furthermore, the mechanical phenotype of the cell can be used to analyze the stability and deformation of the internal structure of the cell, thus providing good guidance for the genesis and proliferation of cancer cells.

In addition, there are many other techniques for measuring the mechanical properties of single cells, such as the magnetic twisting (MT) [154] and microarray methods [155]. The principle of the bead torsion method is to design a predetermined magnetic field to move the magnetic particles in the magnetic field according to the planned path. We combined the magnetic beads with the cell surface after binding a certain polypeptide and made the cell rotate and move through the force generated by the magnetic field. Daniel [156] used the adhesion force in the living cell and the mechanical properties of the cytoskeleton for evaluation. Chen [157] designed a magnetic twisting instrument with feedback control. The measurement frequency could reach 1 kHz, and this instrument effectively characterized the mechanical properties of cardiomyocytes. The microarray method, also known as the microcolumn method, can obtain the local traction, multicell force, and the mechanical behavior of cells under external stimulation. The microcolumn in the microarray is made of flexible silicone resin, which is a cantilever structure with vertical distribution according to a certain array. The cells to be tested can adhere to the top of multiple microcolumns. When the cells expand or compress, the microcolumns will bend to a certain extent, so that the mechanical information can be evaluated. Kelvin [158] used a microarray to evaluate and improve the contractile function of cardiomyocytes. The mechanical properties technology mentioned above has opened a convenient door for us to explore the mechanical universe of cells and the mechanism of cell mechanics. All in all, the cell mechanical model can help us to explain the measured experimental parameters, and they can support each other. For example, the measured parameters can calibrate the constitutive properties of the mechanical model and improve the mechanical model. In addition, if we only rely on measurement technology, we cannot clearly explain the complete mechanical properties of cells, nor can we explain the influence of the internal biomechanical process on the mechanical behavior of cells.

## 4. Application Direction

Important biological information is contained inside and outside the cell. The mechanical properties of different cells are different, and the mechanical properties of different stages of the same cell will also change significantly. Information is exchanged between the cell and the outside environment all the time. The cytoskeleton inside the cell constantly polymerizes and depolymerizes, and all kinds of motion proteins spread freely. With the established cell mechanics model and various experimental techniques, we analyze the mechanical properties and behavior of cells so as to reveal the mystery of life. The application direction of cell mechanics mainly includes the following three areas: disease diagnosis and treatment, regeneration and repair of biological tissue, single cellular micromanipulation, and other applications.

### 4.1. Disease Diagnosis and Treatment

Biological cell mechanics is a new research field, and it has important research and application value in the research of human diseases [159]. Studying human diseases from cellular and molecular levels is helpful in uncovering the pathological mechanism behind diseases on a deeper level. Diseases not only change the biological function of cells but also lead to the abnormality of physical and structural characteristics of cells. At present, most of the research is mainly on molecular, biological, and pathological aspects, but there is little on cell mechanics. The pathological characteristics of organisms depend on the cells that make them up. The variation of tissue structure and mechanical characteristics of single cells is the basis of the occurrence, development, and prognosis of all diseases.

In the field of medicine, some cancers refer to the malignant tumor, which originates from epithelial tissue. It is the most common type of tumor, and the diagnosis and treatment of cancer are very difficult. Although a variety of carcinogenic factors have been identified, it is difficult to detect the formation of cancer cells in time due to the “zero time” characteristics of cancer occurrence. As the generation, development, and metastasis of cancer cells are usually accompanied by changes in microfilaments and microtubules in the cells, in return, these changes will also transform whole-cell mechanics. In order to solve the problem that cancer cells are difficult to recognize, Makarova [160] used atomic force microscopy to detect the elastic modulus of premelanoma and melanoma cells and found that the elastic modulus of precancerous cells was lower than that of normal cells. On the basis of this difference, researchers also used fluid shear force [17,161,162,163] to detect cancer cells. By designing a microfluidic chip, we can observe the size of the deformation of cells due to shear force and established the viscoelastic mechanical model to determine whether cancer cells occur. Pablo [164] combined Raman spectrum analysis, the cell mechanics model, and experimental technology to monitor rectal cancer cells. By comparing the components of hundreds of cell lines, we can classify primary and secondary tumor cells and then distinguish different disease stages of cancer, which can be used as a diagnostic tool of cell phenotypes in clinical practice.

Dysgenesis disorder has become a problem of people’s quality of life. With the development of assisted reproductive technology, this problem has been improved to some extent. Nowadays, the research on assisted reproductive technology focuses on the improvement of the success rate. Both Matsuura and Koike [165,166] have designed a dynamic culture device that can swing periodically. The flow of the culture medium is used to generate shear force to stimulate the cells. The research shows that the survival rate and development speed of the embryos in dynamic culture are significantly improved. After that, the development of a microfluidic chip as a tissue chip of embryo culture and the fallopian tube was inspired by the shear force of fluid. Chang [167] designed a microfluidic chip to simulate the human uterus, in which the inner wall of the structure used to culture embryos is designed to be concave and convex so that the culture medium can produce irregular internal flow to stimulate embryos with the shear force of the fluid. Huang [168] made the droplets containing embryos move on the microfluidic platform by using electrowetting on dielectrics so that the fluid movement inside the droplets produced shear force on the embryos. Compared with static culture, it was found that the survival rate and culture speed of the embryos were improved. Experimental results have shown that the incubation rate was 24% higher in dynamic culture than in static culture.

Smooth muscle is the muscle tissue of nonstriated muscle. The ciliary muscle and iris are distributed in the walls of human arteries and veins, the bladder, the uterus, male and female reproductive tracts, the digestive tract, the respiratory tract, and the ciliary muscle and iris of the eye. The abnormal function of smooth muscle will lead to corresponding organ dysfunction and even disease. Irregular connective tissue in the vaginal wall is the main cause of pelvic diseases after delivery. Ferreira [169,170] verified through comparative experiments that loxl1 (lysyl oxidaselike1) could reduce the stiffness and surface adhesion of smooth muscle cells in the vaginal wall, thus reducing the ability of the vagina and pelvic floor to support the pelvic organs, leading to pelvic organ prolapse and causing a variety of gynecological diseases. Atherosclerosis is the main cause that may lead to coronary heart disease, cerebral infarction, and peripheral vascular disease. Studies have shown that many macrophages actually originate from smooth muscle cells [171]. Sanyou [172] found that cholesterol depletion mediated by statins may coordinate the migration and adhesion of arterial smooth muscle cells to different ECM proteins and also regulate cell stiffness and cytoskeleton orientation, thus affecting cell biomechanics and having a good therapeutic effect on high cholesterol or cardiovascular disease.

### 4.2. Regeneration and Repair of Biological Tissue

**Figure 6:**
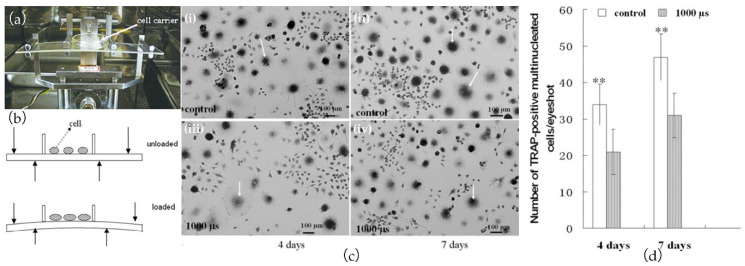
(**a**) Four-point bending device for mechanical loading. (**b**) Schematic diagram of cell stretching with or without load. (**c**) RAW264.7 cells and TRAP-positive multinuclear cells (white arrows) after 4 and 7 days loading (1000 μs, 1 Hz). (**d**) The number of TRAP-positive multinuclear cells. After 4 and 7 days of mechanical loading, the number of TRAP-positive multinuclear cells decreased, indicating that mechanical loading inhibited the osteoclast differentiation of RAW264.7 cells. (Adapted by kind permission from [173]).

Biological tissue is a substance between cells and organs, which is generally composed of many cells and intercellular substances with similar morphology and function. Biological tissue consists of epithelial tissue, connective tissue, nerve tissue, and muscle tissue. Appropriate external stimulation will have a great influence on cell activity and function in the tissue, promote cell proliferation and differentiation, and thus affect tissue homeostasis and the physiological response. We take bone tissue in the connective tissue as an example. Bone tissue is a common biological tissue and also the hardest tissue in human and animal bodies. Bone tissue is composed of osteoclasts and osteoblasts. Normal bone is the result of the dynamic balance between osteoclast absorption and osteoblast reconstruction. The osteoclast is a kind of multinucleated giant cell that is composed of several monocytes and fused in many ways. According to the research, the mechanical environment [173] has an important impact on maintaining the stable morphology and normal work of bone tissue, and appropriate external mechanical stimulation is of great significance for bone reconstruction, as shown in Figure 6. The activity of bone cells [174] is closely related to the formation of bone, and external mechanical stimulation is helpful to enhance the activity of osteoblasts, which is also of great significance for recovery from fractures. Many researchers have used biomechanical devices to simulate the external mechanical environment of cells, such as extrusion pressure, stretching stress, tensile stress, and fluid shear stress [175,176,177]. They established the three-dimensional cortical skeleton combined model based on the finite element method to simulate the mechanical behavior of cells and explore the response mechanism of bone cells to the external mechanical environment under different stress modes. The study showed that the magnitude and frequency of external stress and the concentration of parathyroid hormones are also important for the activity of bone cells.

In addition, mesenchymal stem cells are widely present in bone marrow and have a strong ability to self-replicate. The mesenchymal stem cell (MSC) is a kind of pluripotent stem cell that has nearly all the characteristics of stem cells, which have the ability of self-renewal and multidirectional differentiation. It is clinically used to solve a variety of blood system diseases, cardiovascular diseases, cirrhosis, nervous system diseases, knee injury repair, autoimmune diseases, and so on. MSC plays a crucial role in bone remodeling and formation. Osteoblasts are derived from the differentiation of MSCs, and the mechanical stress of MSCs affects the differentiation rate of cells [178,179]. In addition, MSCs are also used in plastic surgery and reconstruction surgery. Their lipogenic potential should be strictly controlled to avoid their differentiation into adipose tissue. Jorge [180] used mechanical regulatory protein YAP (Yes-associated protein)/TAZ (tafazzin) to participate in the differentiation of MSCs and inhibit the formation of adipose tissue.

### 4.3. Single Cellular Micromanipulation

Due to the progress of micromanipulation technology, many experimental techniques have been developed to operate single cells and detect the mechanical response of cells. The introduction of the cell mechanics model can describe all kinds of mechanical behavior changes of cells in micromanipulation, including cell injection, cell stretching, and cell cutting. Taking cell injection as an example, microinjection is an important technology to introduce foreign substances into cells. However, little is known about the response mechanism in the process of cell injection, and the cell mechanical model can effectively characterize the mechanical properties of cells. Tan [181,182] proposed a linear elastic solid model based on the membrane theory, established the quasistatic equilibrium equation between the injection force and the deformation of biological cells, and solved it with the Runge Kutta numerical method. By using this model, the deformed cell membrane shape, in the process of microinjection, can be obtained. Through this model, we can also infer the distribution of membrane stress and tension, cell deformation, and internal cell pressure in the case of syringes with different radii.

**Figure 7:**
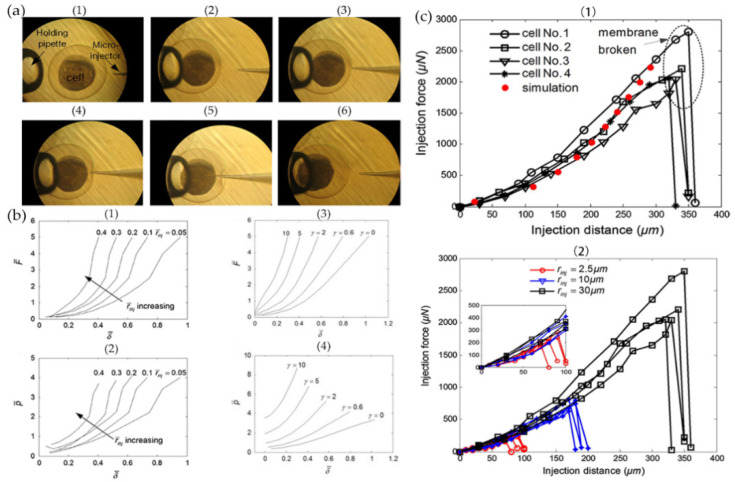
Injection model. (**a**) (1)–(6) Zebrafish cell injection experiment. (**b**) (1),(2) The injection force $\bar{F}$ and cell internal pressure $\bar{P}$ vary with the injection distance $\bar{\delta }$ under different injection radii; (3),(4) the injection force $\bar{F}$ and internal pressure $\bar{P}$ vary with the injection distance $\bar{\delta }$ under different materials. (**c**) (1) Experimental data of injection force and injection distance under 30 μm injection radius when membrane of different cells break; (2) experimental data of injection force and injection distance under different injection radii when membrane of cells break. (Adapted by kind permission from [170]).

In order to study the mechanical response of adherent cells during microinjection, Shen [183] proposed a hyperelastic cell mechanical model and analyzed the distribution of force and stress between adherent cells during microinjection, as well as the deformation of the cell membrane. Liu [184] proposed a mathematical injection model. The cells were modeled by a hyperelastic membrane and cytoplasm. The Lagrange multiplier and Rayleigh–Ritz technology were used to minimize the potential energy of cells. Then, they established a cell equilibrium model and studied the effects of injection force, injection distance, radius of microinjector, and membrane stress on the mechanical behavior of cells, as shown in Figure 7. Furthermore, Liu [47] also studied the damage degree of zebrafish cells in the process of microinjection based on the dissipative particle dynamics model. By studying the number of broken chemical bonds in cells, we can simulate the damage of cells; the more the number of chemical bond breaks, the greater the damage degree of the cells.

Yan [185] established a three-dimensional cell solid model to analyze the stress and deformation of cells when they are subjected to large-scale mechanical loads. The proposed cell damage criteria and stochastic simulation technology linked the mechanical load and cell damage through mechanical methods and predicted the degree of cell damage induced by the load. Kim [186] stained mammary gland epithelial cells with calcein and then analyzed the cell deformation and lysis under compression according to fluorescence intensity change.

### 4.4. Other Applications

In addition, the study of cell mechanical properties has also been used in sanitary sterilization. The research on cell mechanics has also been applied to the related treatment of bacteria, which has been applied in food sterilization, sanitary product sterilization, and waste sterilization. When the hydrostatic pressure is large (>50 MPa) [187], the bacteria undergo great physiological changes or die. In the food industry, hydrostatic pressure treatment of 50–100 MPa is often used to achieve the sterilization effect [188].

**Figure 8:**
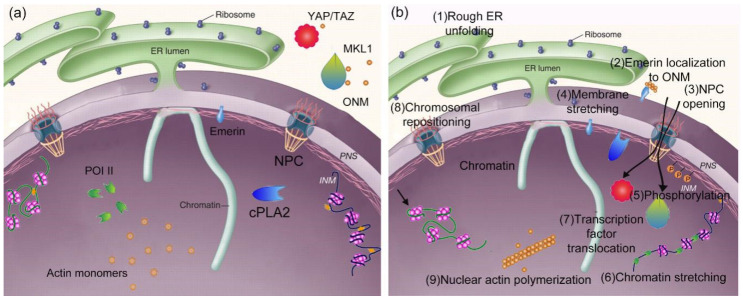
Schematic diagram of the mechanism of nuclear response to mechanical stimulation. (**a**) Under normal conditions, the internal and external environment of the nucleus. (**b**) After mechanical stimulation, the response of the internal and external environment of the nucleus. ((1) Stretching of nuclear membrane can change the conformation of rough endoplasmic reticulum. (2) Applying force to promote the transfer of emerin from INM (inner nucleus membrane) to ONM (outer nucleus membrane) to regulate chromatin tissue. (3) Nuclear membrane stretching may introduce nucleoplasmic phospholipase (cPLA2) into INM. (4) Increasing membrane tension can adjust NPC (nuclear pore complex) permeability. (5) Force transfer to the nucleus results in post-translational modifications and kinetic changes of lamin A/C and INM proteins. (6) External forces can cause chromatin stretching and change the accessibility and activity of transcription factors and enzymes. (7) The opening and isolation of nuclear pores at the nuclear membrane can regulate the location and activity of transcription factors.)

At the same time, the mechanical properties of cells are also related to the mechanical signal transduction inside the cells. When cells are stimulated by external forces, integrin, as a mechanical sensor on the surface of cells, can transmit external mechanical signals to the cytoskeleton, thus regulating the mechanical behavior of the skeleton. When the external domain of integrin is combined with extracellular matrix protein, the configuration of the internal domain of integrin will change, and, finally, the extracellular matrix and intracellular skeleton will be synthesized as a whole; tension fibers and adhesion spots are also generated. In addition to regulating the mechanical properties of cells through transmission, mechanical signals can also transform external mechanical signals into chemical signals through a transduction mode. Cheng [189] deeply studied how mechanical signals on cell membranes can be transformed into biochemical signals and molecular mechanisms under different stiffness of the cell matrix so as to realize the targeted treatment of related diseases. In addition, because of the construction of microtubules and microfilaments in the skeleton model, the simulation of the net-like objects is realized. In the meantime, the expression of genetic material will change when the nucleus is stimulated mechanically. Therefore, according to the microfilaments and microtubules in the model, mechanical stimulation outside the cell membrane can be transmitted to the inside of the nucleus, thus affecting the expression of genetic material [190]. According to these differences, the nucleus can be used to sense the mechanical stimulation of cells. A schematic diagram is shown in Figure 8.

## 5. Concluding Remarks

For the future of the cell mechanics model, I reckon that the model should satisfy the following conditions: (1) The cell model needs to be consistent with cell structure, including the cytoskeleton, crosslinked protein, and functional protein; (2) the model should be in line with the mechanical properties of the cell in reality, such as the elastic behavior of the cell, the viscoelastic properties of the nucleus, and the polymerization and depolymerization of the cytoskeleton; (3) the cell model to be established should be applicable and can simulate different cell types and analyze the stress and deformation of cells under different loads; (4) it can be closely combined with molecular dynamics to analyze the mechanical behavior of cells from the molecular perspective. In general, all the cellular mechanical models mentioned above have partial explanations for some of the mechanical properties of cells. These mechanical properties can reflect the degree of cell health, which provides a reference for the diagnosis and treatment of some human diseases. However, there is no good explanation of the relationship and the scientific mechanism between human diseases and the mechanical model. In my opinion, with the continuous improvement of the mechanical model and the continuous fusion of new theories and advanced measurement methods, the cellular mechanical model will make a greater contribution to human health.

## Figures and Tables

**Table 1:** Main components of cells.

Type	Description	Composition
membrane	Elastic semipermeable membrane composed of phospholipid.	Phospholipid bilayer, glycoprotein, glycolipid.
cytoplasm	A general term for all translucent, colloidal and granular materials outside the nuclear zone.	Golgi apparatus, mitochondria, endoplasmic reticulum, ribosome.
nucleus	The main site of storage, replication, and transcription of genetic information in cells.	Nuclear membrane, chromatin, nucleolus, nuclear matrix.
cytoskeleton	The network structure of protein fibers.	Microfilaments, microtubules, intermediate fibers.

**Table 2:** The comparisons of cell mechanical models.

Name	Type	Characteristic	Application
Continuous cortical membrane model	Droplet model	A fluid-like sphere surrounded by continuous cortex	Suspension cells
	Solid model	Isotropic solid substances	Suspension and adherent cells
	Damping model	A frequency-domain model under dynamic forces	Adherent cells
	Two-phase model	The solid–liquid duality	Articular chondrocyte
Discrete network skeleton model	Tension integration model	A space balance system	Adherent cells
	Porous solid mode	A regular network model	Adherent cells
	Cable net model	An ideal articulated cable network	Red blood cells
	Biochemical mechanical model	Combination of biochemical process and mechanics	Adherent cells
Cortical skeleton combined model	Based on finite element	From the point of view of finite element	Suspension and adherent cells
	Based on molecular dynamic	From the point of view of molecules	Suspension and adherent cells

## Appendix A

**Table A1:** Main biomechanical parameters and common formulas.

Biomechanical Terminology	Explanation	Common Formula or Law	Reference
Elasticity	Elasticity refers to the property that an object can recover its original size and shape after deformation.	△F = −k·△x, when the object deforms, the elasticity is directly proportional to the elongation. (Hooke’s law)	[14,19,39,40,43]
Viscosity	The resistance of fluid to deformation under shear stress is measured.	τ = µ$\frac{du}{dy}$refers to the velocity gradient along the Y direction, μ is the viscosity coefficient.	[14,54,55,56,58,61,65,69,82,83,109,110,123,143,151,155]
Viscoelasticity	The comprehensive properties of viscosity and elasticity of fluid	${\gamma \;=\;\gamma }_{E}{+\gamma }_{H}{+\gamma }_{Y}$ γ *:* total deformation; $\gamma_{E}$: ordinary deformation; $\gamma_{H}$: the delayed high elastic deformation;$\;\gamma_{Y}$: viscous deformation.	[6,18,19,63,112,113,143]
Stress (σ)	Internal force per unit area	σ = P/A, ratio of load to section area.	[15,34]
Shear stress	The interaction force between two sides of any section (shear plane)	τ = $\frac{F_{S}}{A},$ load $F_{S}$ is parallel to the section.	[13,17,43]
Strain (ε)	The local relative deformation of the object under the action of external force and non-uniform temperature field.	$\varepsilon =\underset{L\to 0}{\lim}\left(\frac{\Delta L}{L}\right)$, L is the original length and ΔL is the elongation.	[42]
Shear strain	The relative shape variable produced by the object during shearing	γ =tanθ, θ is the skew angle; when the shear strain is infinitely small, γ = θ.	[44,85]
Elongational strain	The ratio of the change of the line length to the original line length.	The strain produced by an object in tension or compression.	[42,68,84,124,125,148]
Young’s modulus (E)	A physical quantity describing the ability of solid materials to resist deformation.	E = σ/ε, the ratio of stress to strain.	[7,17,18,38,39,43,48,69,70,105,106,107,123,125,126,133,134,139,140,141,142,143,149,152,155]
Shear modulus (G)	Shear stress characterizes the material’s ability to resist shear strain.	G = τ/γ, the ratio of shear stress to shear strain.	[48,104,110]
Poisson’s ratio(v)	The ratio of the absolute value of the transverse positive strain to the axial positive strain.	Load in elastic range:$\;\varepsilon_{x}=-v\varepsilon_{y}$, v is a constant, beyond the elastic range, v increases with the increase of stress until 0.5; Relations between E, G, v:G=E\[2*(1+v)]	[15,16,55]
Dynamic viscoelasticity	The viscoelasticity of objects in vibration.	It describes the ratio of stress to strain of an object under dynamic load	[51,52,61]
Storage modulus (G’)	The measurement of energy storage in the process of strain cycling and is usually expressed as the real part of the complex modulus.	Complex modulus: G =$\;G^{\prime }$+ jωG” Storage modulus: $G^{\prime }=G*\cos\left(\delta \right)=\left(\sigma_{0}/\gamma_{0}\right)*\cos\left(\delta \right)$	[88,151]
Loss modulus (G’’)	The degree of energy loss when the material deforms; usually expressed as the imaginary part of the complex modulus.	Loss modulus: $G”=G*\sin\left(\delta \right)=\left(\sigma_{0}/\gamma_{0}\right)*\sin\left(\delta \right)$	[88,151]
Loss tangent(tanδ)	Reflect the ratio of viscosity and elasticity of material	Loss tangent: tanδ = $G”/G^{\prime }$	[88,151]

**Table A2:** Characteristics of measurement technologies.

Tool	Measuring Force Range	Object Size
Micropipette aspiration	0.1–$10^{3}$ nN	1–$10^{2}$ µm
Microfluidic technology	1–$10^{3}$ pN	1–$10^{3}$ µm
Optical tweezers technology	0.01–$10^{3}$ pN	10–$10^{5}$ nm
Atomic force microscope	10–$10^{7}$ pN	1–$10^{5}$ nm
Magnetic twisting	1–100 pN	0.1–100 µm
Microarray method	1–100 nN	1–$10^{3}$ µm
